# The potentials and challenges of using fermentation to improve the sensory quality of plant-based meat analogs

**DOI:** 10.3389/fmicb.2023.1267227

**Published:** 2023-10-04

**Authors:** Hosam Elhalis, Xin Yi See, Raffael Osen, Xin Hui Chin, Yvonne Chow

**Affiliations:** ^1^Singapore Institute of Food and Biotechnology Innovation (SIFBI), Agency for Science, Technology and Research (A*STAR), Singapore, Singapore; ^2^Food Science and Technology, School of Chemical Engineering, The University of New South Wales, Sydney, NSW, Australia

**Keywords:** meat alternatives, novel foods, sustainable foods, consumer acceptance, functional starter cultures

## Abstract

Despite the advancements made in improving the quality of plant-based meat substitutes, more work needs to be done to match the texture, appearance, and flavor of real meat. This review aims to cover the sensory quality constraints of plant-based meat analogs and provides fermentation as a sustainable approach to push these boundaries. Plant-based meat analogs have been observed to have weak and soft textural quality, poor mouth feel, an unstable color, and unpleasant and beany flavors in some cases, necessitating the search for efficient novel technologies. A wide range of microorganisms, including bacteria such as *Lactobacillus acidophilus* and *Lactiplantibacillus plantarum*, as well as fungi like *Fusarium venenatum* and *Neurospora intermedia*, have improved the product texture to mimic fibrous meat structures. Additionally, the chewiness and hardness of the resulting meat analogs have been further improved through the use of *Bacillus subtilis*. However, excessive fermentation may result in a decrease in the final product’s firmness and produce a slimy texture. Similarly, several microbial metabolites can mimic the color and flavor of meat, with some concerns. It appears that fermentation is a promising approach to modulating the sensory profiles of plant-derived meat ingredients without adverse consequences. In addition, the technology of starter cultures can be optimized and introduced as a new strategy to enhance the organoleptic properties of plant-based meat while still meeting the needs of an expanding and sustainable economy.

## 1. Introduction

Plant-based meat analogs are created to meet consumer demand and provide a sustainable protein supply in the future. They have a nutritional profile almost identical to real meat and are considered excellent sources of protein (Bryngelsson et al., 2022). Furthermore, they were manufactured to mimic various types of meat products in texture, color, nutritional value, and flavor (Choudhury et al., 2020). Tempeh and tofu products made from soybeans are well-known traditional plant-based protein products, which have been consumed for centuries mainly in Asian countries (Joshi and Kumar, 2015). However, the global demand for sustainable diets to replace real meat products, as well as other environmental consequences, has pushed the industry’s focus on developing meat analogs. The global market of meat alternatives is estimated to grow at a compounded annual growth rate (CAGR) of 7.2% and reach USD 15.6 billion by 2026 (van Vliet et al., 2020). A plant-based meat analog contains mainly proteins, fats, adhesives, colorings, and flavorings. Various protein types such as soybeans, peas, and wheat are often blended and subsequently, the meat-like fibrous textures are developed through multiple techniques, including extrusion, shearing, spinning, and 3D printing (Dekkers et al., 2018a; Sha and Xiong, 2020). Coloring agents are an essential component of meat alternatives to resemble real meat appearance. Heat-stable pigments such as cumin, turmin, and carotene are the most prevalent in uncooked meat, while heat-labile colorants and reducing sugars are often used to resemble cooked meat appearance (Kyriakopoulou et al., 2019; Bakhsh et al., 2021). Savory spicing, meat, and savory aromas, and their precursors, are currently used as flavoring agents (Kyriakopoulou et al., 2021).

Animal welfare, environmental ethics, and health benefits are the major advantages of consuming plant-based diets (Bryant et al., 2019). But, if plant-based meat alternatives cannot take over the associated eating experiences of traditional meat products, then reductions in their acceptability can be expected. This might explain why their absolute market share remains low, accounting for only about 1% of the total meat market (Choudhury et al., 2020). One of the major barriers to transitioning to plant-based meat analogs is their low sensory quality (Hoek et al., 2011; Onwezen et al., 2021). For example, extrusion as the mostly current way to create texturized protein used in plant-based meat analogs, resulted in a fibrous network that does not completely resemble the structure of muscle fibers (Ullah et al., 2019; Sha and Xiong, 2020). Plant-based meat substitutes are often perceived as dry or lacking in juiciness, as well as having poor appearance. Furthermore, off-flavors such as bean flavor, bitterness, aftertaste, and astringency are commonly reported with plant-based alternatives (Gläser et al., 2020; Sha and Xiong, 2020; Wang et al., 2022). Recently, the use of genetically modified microorganisms as additives has also raised public concerns (Dekkers et al., 2018a; Fraser et al., 2018; Imran and Liyan, 2023).

The scientific literature on the studies that find sustainable and novel approaches to modulate the plant ingredients used in meat alternatives and improve the final product’s sensory quality is limited. Fermentation is a traditional technique that has been used to preserve perishable foods and improve their nutritional, and sensory quality. It has been reported to improve various product textures, taste, aroma, and appearance in an economical and energy-efficient way (Gänzle, 2009; Sakandar et al., 2020). With today’s advanced technology, it is possible to control the fermentation process more efficiently and environmentally friendly by selecting microorganisms, including bacteria and fungi (starter cultures). The use of fermented plant materials and designing starter culture technology in the production of plant-based meat analogs has received little attention, despite its potential to enhance their sensory quality. This review focuses on an in-depth examination of how fermentation influences the sensory quality of plant-based meat analogs. Initially, it presents an overview of current scientific knowledge regarding the disadvantages of plant-based meat analogs in terms of sensory quality. As a consequence, the second section of the review examines how fermentation and starter culture technology can be applied to push these boundaries in a more cost-effective and sustainable manner. Lastly, the review presents general recommendations for selecting suitable microorganisms and designing starter cultures, as well as future directions. This review shows the potential of including controlled fermentation and fermented ingredients as a novel approach to improve the sensory quality of plant-based meat analogs.

## 2. Sensory characterization of plant-based meat analogs and the need for new approaches

For the purpose of this review, real meat can be either fresh (uncooked whole-muscle meat, such as boneless steaks), cooked (i.e., cooked beef roast), or fermented meat (i.e., fermented sausages). Plant-based meat alternatives are a broad category of foods that are similar in texture, appearance, flavor, and nutritional quality to real meat products in the human diet. Sensory and nutritional quality are the key drivers for consumer acceptance of plant-based meat analogs (Fiorentini et al., 2020; Boukid, 2021). Sensory quality is the human response to stimuli experienced with food and can generally be described using terms of appearance, flavor, and texture (Drake and Delahunty, 2017). The sensory characteristics of the meat analogs are mainly affected by the product’s components, formulation, and structuring technology (Van der Weele et al., 2019). Details regarding components, formulation, and structuring technologies of plant-based meat analogs are outside of this review scope and have been reviewed recently in depth by other authors (Fresán et al., 2019; Samard and Ryu, 2019; Fiorentini et al., 2020; Boukid, 2021; Sun et al., 2021; Younis et al., 2022; Flint et al., 2023; Imran and Liyan, 2023; Yang et al., 2023). Hereby, the most related data was briefly highlighted to explain the current state of the sensory characteristic of resulting products and demonstrate the urgent need for a novel approach to increase their acceptability.

**Figure 1:**
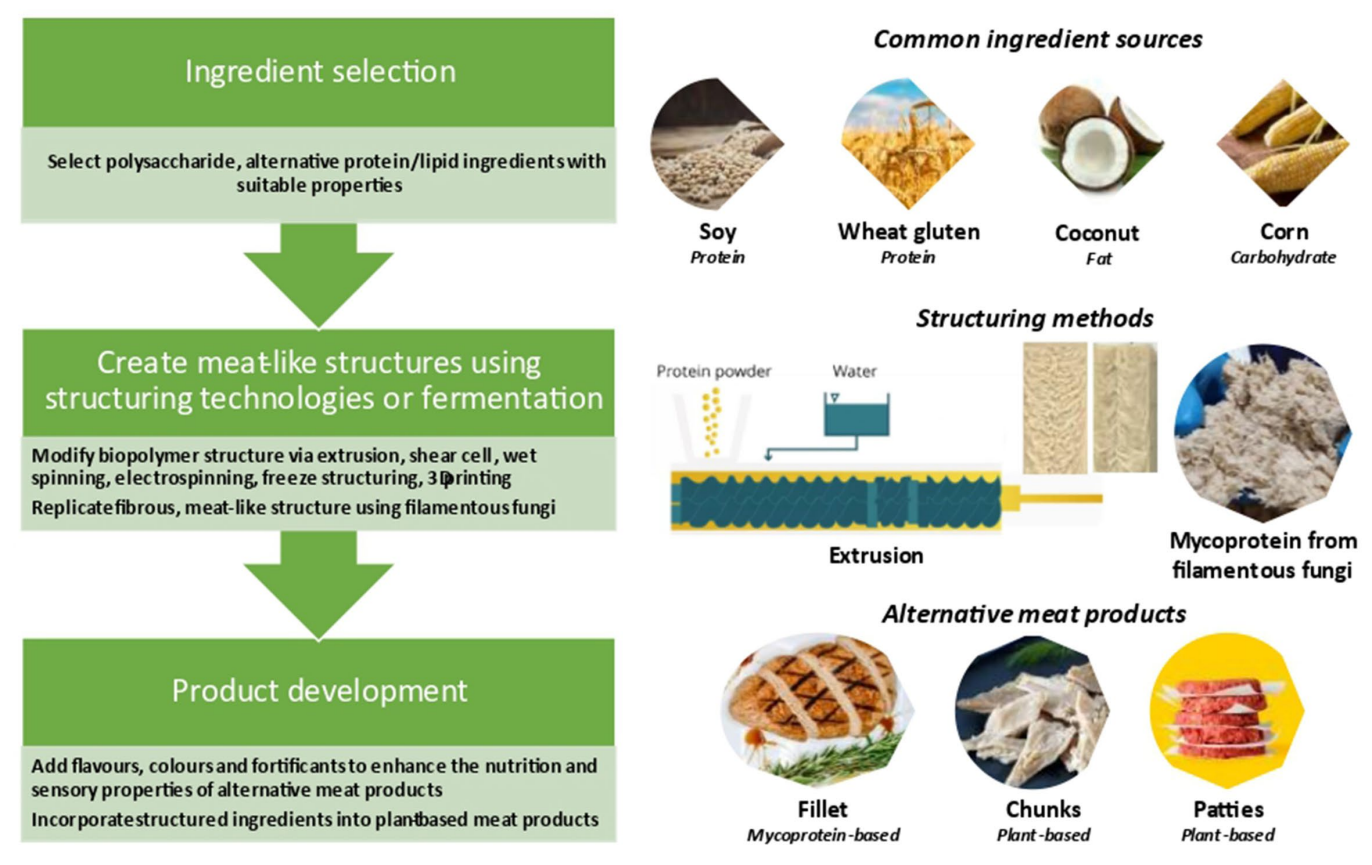
Main ingredients and processes employed to create plant-based meat analogs [adapted from Impossible burger (2018), Planted (2019), Pietsch et al. (2021), The Better Meat Co (2021, 2023), and Good Food Institute (2022)].

As mentioned earlier, Plant-based protein foods such as tempeh and tofu are commonly consumed in Asian countries and the main aim of creating plant-based meat analogs is to increase their acceptability worldwide. A broad range of ingredients can be used for of plant-based meat analogs, including proteins (e.g., soy, other legumes, wheat gluten), carbohydrates (e.g., wheat, corn, and potato), and lipid (soy oil, sunflower oil, corn oil, rape seed oil, palm oil, and coconut oil/butter; Fu et al., 2023). Furthermore, minerals such as Fe, Zn, and Na, as well as vitamin B12, and other ingredients including binding agents (e.g., xanthan gum, carrageenan, and methylcellulose), flavors (e.g., spices and herbs) and (e.g., red cabbage, red beets, and leghemoglobin) are commonly added. These materials are carefully selected, mixed, and structured to mimic real meal products (Wild et al., 2014). Figure 1 shows the main ingredients and processes employed to create plant-based meat analogs. Extensive attempts have been made to improve the sensory quality of plant-based meat analogs, but several drawbacks are still reported as discussed below.

### 2.1. Flavor

A recent survey showed that consumer acceptance of plant-based meat analogs was lower in the USA compared to Asian countries, mainly India and China (Bryant et al., 2019). This low consumer acceptance is likely related to the poor sensory properties of plant-based meat products. Overall, most plant ingredients do not have any perceptible meaty aroma or taste (Fuhrmeister and Meuser, 2003; Taherian et al., 2011). Furthermore, off flavors including green, beany, astringent, bitter, and metallic tastes are also considered potential constraints in some plant ingredients, such as legumes (Leonard et al., 2022). Specifically, off-flavor volatiles such as n-hexanal, ethyl vinyl ketone, and 1-octen-3-ol are mainly formed by the oxidation of unsaturated fatty acids (Frankel, 1991; Wang et al., 2022), whereas non-volatiles linked to off tastes were due to other substrates like phenolic compounds, free fatty acids, bitter amino acids, saponins and alkaloids (Campos-Vega et al., 2010). During lipid oxidation, unsaturated fatty acids form primary oxidation products hydroperoxides, which then react with free radicals to form a wide variety of off-flavor secondary compounds (Schaich, 2017). According to Gläser et al. (2020), pea protein contained a number of lipid oxidation products that were linked to the bitter taste of the resulting plant-based meat analogs. These products included trihydroxy octadecenoic acid, hydroxy octadecadienoic acid, −linolenic acid, linoleic acid, and 2-hydroxyoleic acid (Gläser et al., 2020). The presence of such compounds negatively impacts the formation of meaty flavors from plant-based meat ingredients (Li and Li, 2020). Limited success has been reported with applying flavorings to mask the off-flavors or replicate the meaty flavors (Fiorentini et al., 2020). Additionally, some flavoring agents may be destroyed during cooking and have adverse health effects (Li and Li, 2020). More specifically, during thermal processing, carbon nanostructures are formed due to the polymerization or complexation of partially hydrolyzing proteins, lipids, and other macromolecules with synthetic flavorings. Such unknown nanostructures cause negative physiological impacts or sever health risks including mutagens, carcinogens, and teratogens (Sharma et al., 2018; Blázquez et al., 2019). In recent years, recombinant proteins such as leghemoglobin (iron-rich heme) have been used successfully to release the characteristic flavor of meat during cooking (Dekkers et al., 2018a; Fraser et al., 2018). However, the use of recombinant proteins still raises public concerns because of potential health and environmental risks that limit it application in foods (Fraser et al., 2018).

### 2.2. Texture

Extrusion, shear cells, freeze structuring, wet spinning, and electrospinning are the most reported texturized methods to produce plant-based meat analogs with promising results (Osterhaus, 1978). However, their applications present its challenges, and success in producing fibrous meat-like structures in a cost-efficient way has been limited. First, it requires highly soluble forms of protein, and in some circumstances, only refined sources of protein can be used. The proteins have to undergo preconditioning processes such as hydration, defatting, and in some cases, blending with other ingredients (i.e., pectin and starch) to improve their functionality (Crowe and Johnson, 2001; Dekkers et al., 2018a,b). Additionally, several parameters have to be closely monitored including barrel temperature, pressure, and feed rates of powder/water affecting the cost, productivity and variability (Rehrah et al., 2009; Garg and Singh, 2010; Guerrero et al., 2012; Basediya et al., 2013). At the end, overheating could occur during the texturizing process, resulting in poor quality texturized products (Rueda et al., 2004; Kyriakopoulou et al., 2019). The resulting texturizing proteins have been reported to have a more spongy structure than a fibrous meat-like structure in some cases (Monteiro et al., 2018; Samard and Ryu, 2019). It is also difficult and expensive to scale up certain texturizing technologies (Gu et al., 2020; Younis et al., 2022). At commercial scale, extrusion and shear cell methods are most commonly used because of their robustness and capacity for large-scale production (He et al., 2020).

### 2.3. Color

Another limitation of plant-based meat analogs is their color and appearance. Plant proteins (e.g., soy protein and gluten) typically do not have the red color of raw meat nor the brown color of cooked meat (Kyriakopoulou et al., 2019). Therefore, coloring agents must be added to plant-based meat to mimic real meat’s appearance. Heat-stable coloring agents such as annatto, caramel, and carotene are used in plant-based meat analogs to mimic the red color of raw meat. However, these coloring agents cannot replicate the color of the cooked meat (Rolan et al., 2008; Malav et al., 2015; Ajwalia, 2020). It may remain red after cooking, making it difficult for consumers to determine the meat analog’s degree of doneness. Moreover, overheating can cause some coloring agents to become yellow (Bohrer, 2019; Kyriakopoulou et al., 2019; He et al., 2020; Lee et al., 2020; Sakai et al., 2022). Heat labile color compounds, such as beetroot extracts and betanin, can mimic both raw and cooked meat color changes, but they are not always effective since meat analogs may not be in the optimal pH range for these extracts to perform the appropriate color shift (Rolan et al., 2008; Kyed and Rusconi, 2009; Kyriakopoulou et al., 2021). Although acidulants such as acetic acid, lactic acid, and citric acid can be used to adjust the pH of the meat analogs, such modifications are still ineffective and associated with poor taste and texture (Downey et al., 2008). Colorants are used with other ingredients such as hydrated alginate, maltodextrin, and ascorbic acid to better preserve the color and limit color migration inside the colored meat analog (He et al., 2020). Lastly, another contribution of the leghemoglobin is to improve the color of meat analogs and covert from red to brown during cooking, but its use is linked to potential health and environmental risks (Torres et al., 2009; Fraser et al., 2018; Boukid, 2021; McClements and Grossmann, 2021). Table 1 summarizes the major ingredients and techniques used in plant-based meat analog manufacture, their positive contribution, and current limitations. Overall, these observations illustrate that the current methodologies to manufacture plant-based meat analogs are not sufficient to meet the requirements and possible alternative ways should be explored.

**Table 1:** Main ingredients and techniques used in plant-based meat analogs production, their positive contribution, and current limitations.

Target	Ingredients	Intended contributions	Challenges	References
Taste and aroma	Natural spices and herbs	Masks beany notes and prevent lipid oxidation	Beany flavor is still present	Fiorentini et al. (2020), Lee et al. (2020), Li and Li (2020), and Wi et al. (2020)
	Maillard reaction precursors (e.g., reducing sugars amino acids)	Produce meaty or roasty volatiles	Flavoring agents may be destroyed during cooking	
	Hydrolyzed vegetable proteins	Impart a meat-like flavor		
	Vegetable oils	Replace animal fat roles in flavor formation	Adverse health effects	
Appearance/color	Annatto, lycopene, turmin, cumin, canthaxanthin	Red meat color for fresh products	Red piments remain after cooking	Ajwalia (2020), Bohrer (2019), Fraser et al. (2018), He et al. (2020), Herbach et al. (2004), Kyriakopoulou et al. (2021), Malav et al. (2015), Rolan et al. (2008), and Schreuders et al. (2019)
	Betanin, beetroot extracts, and soy leghemoglobin	Red to brown color changes during cooking	Difficulty estimating degree of doneness	
	Reducing sugars	Browning during cooking	A yellow color may be produced with excessive heating	
	Leghemoglobin	Has meat-like appearance similar to myoglobin	Adverse health and public concerns	
Texture	Texturizing plant proteins, i.e., extrusion, spinning, shearing and 3D printing	A meat-like fibrous structure synthesis from plant proteins	A spongy structure is still reported	Lin et al. (2002), Rehrah et al. (2009), Garg and Singh (2010), Guerrero et al. (2012), Basediya et al. (2013), and Balestra et al. (2019)
			Several parameters must be monitored during texturizing causing high variability	
	Non-texturizing plant proteins, starch and fibers	Improves water binding and	Change in color due to the Maillard reaction, hydrolysis, caramelization, and degradation of pigments	
	Vegetable oil/butter	Improve mouth feel	Require high temperature, pressure, mechanical energy	
	Transglutaminase	Improves the binding and slice ability by inducing crosslinks between the proteins	High cost	
			Difficult upscaling	

## 3. Fermentation and technical challenges in plant protein-based meat analogs

**Figure 2:**
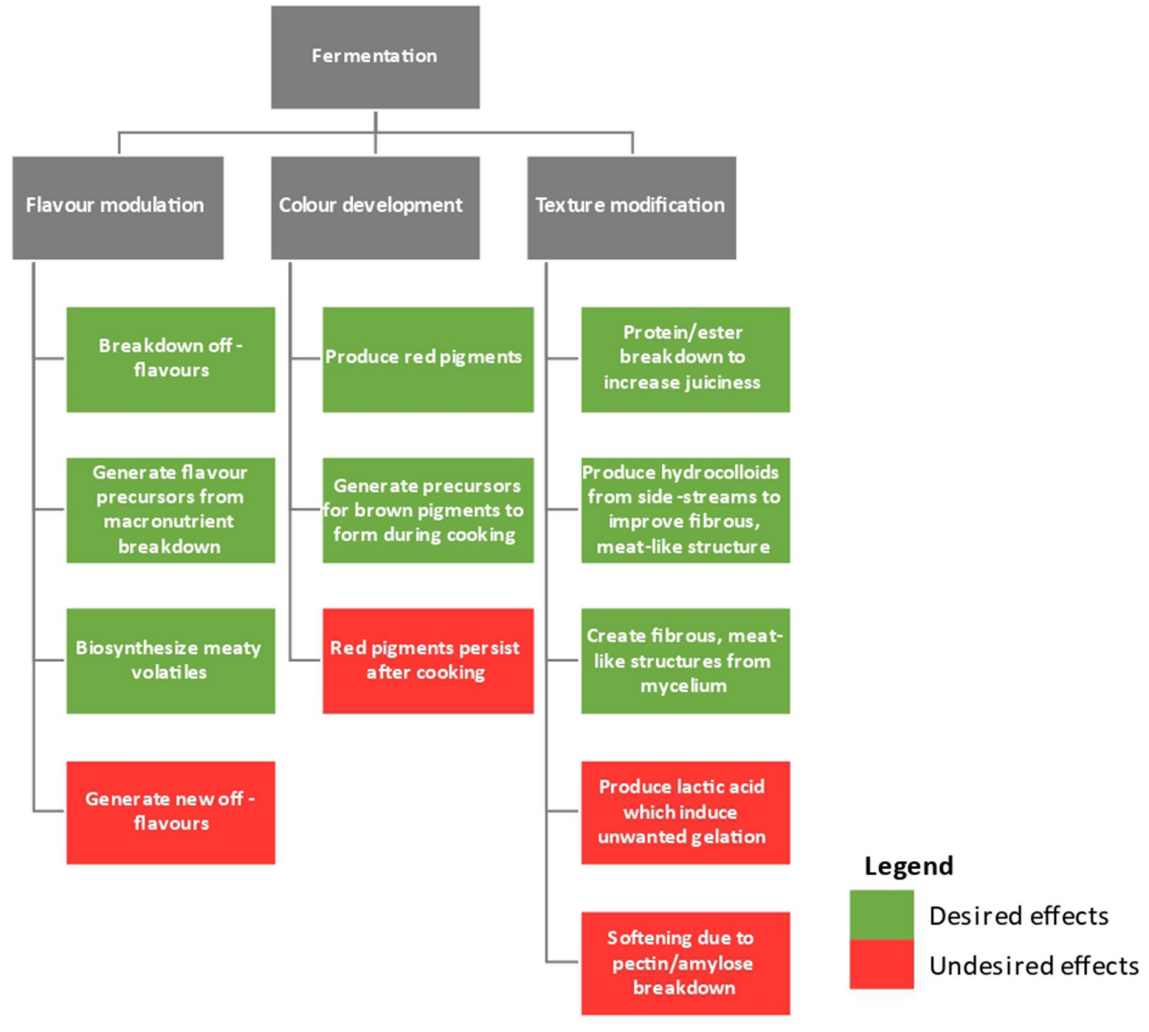
An overview of the impact of fermentation on the sensory quality of plant-based meat analogs.

Meat alternatives should match conventional meat products in terms of texture, flavor, color, and nutrition to be widely accepted by consumers (Michel et al., 2021). Some consumers are wary about the use of additives and multiple processing steps to produce plant-based meat. Specifically, they are concerned that these approaches negatively impact the sustainability, safety, and nutrition of the final product (Kyriakopoulou et al., 2019; Ismail et al., 2020). It is necessary to optimize the quality of raw materials more effectively to reduce the number of additives and processing steps while maximizing the products’ nutritional content (Elhalis et al., 2023c). Fermentation technology might be a novel method for making such adjustments sustainably. Figure 2 shows the overflow contribution of fermentation on plant-based meat analogs sensory quality.

### 3.1. Taste and aroma

Modulating the plant-based meat analogs’ taste and flavor is generally performed by masking the natural off-notes and adding desirable flavoring agents (Wi et al., 2020). Small attention has been paid to the impact of microbial fermentation on the commonly used plant ingredients in meat analogs and their role to improve the final product taste and aroma.

#### 3.1.1. Off-flavor breakdown

Fermentation can be an effective method for reducing off-flavors in plant ingredients. Off-flavor notes (e.g., beany aroma) present in soybean decreased or disappeared after fermentation using *Kluyveromyces marxianus* (Hu et al., 2019), *B. amyloliquefaciens* (Cho et al., 2003; Jiang et al., 2019; Yang et al., 2019; Wang et al., 2021), and *B. subtilis* (Sarkar and Tamang, 1995; Owens et al., 1997; Shrestha and Noomhorm, 2001; Shrestha et al., 2010)*, Weissella confusa* (Tuccillo et al., 2022). These studies demonstrated how fermentation can be used to denature lipoxygenases [lipoxygenase catalyzes the oxidation of polyunsaturated fatty acids to form off flavors like beany notes, mainly aldehydes such as (E)-2-nonenal, hexanal, and (E, E)-2,4-nonadienal] or mask the off-notes present naturally in beans by producing desirable microbial volatiles (Wang et al., 1997; Jiang et al., 2018). For example, a partial denaturation of the lipoxygenase and lipase enzymes was observed during rice bran fermentation by coculture of *B. subtilis*, *Saccharomyces cerevisiae*, and *L. plantarum*, resulting in a decrease in their activities and formation of off-flavor notes (Su et al., 2022). Similarly, Vong and Liu (2018) showed that microorganisms, such as *Lindnera saturnus*, were able to metabolize the undesirable aldehydes, which are produced by lipoxygenase activities, and convert them into desirable ester compounds. This occurred through oxidizing the aldehydes into acids by aldehyde dehydrogenase or reducing them into alcohols by alcohol dehydrogenase. The resulting metabolites can further react with each other or with other microbial metabolites and formed corresponding ester compounds including ethyl hexanoate, hexyl acetate, 3-hexenyl acetate, propanoate and octanoate, ethyl heptanoate, 2-heptenyl acetates. This fermentation increased the total concentration of esters by 70 times compared to the nonfermented sample, reaching approximately 0.17–0.28 mg/g dry weight. These findings illustrate the capability of microorganisms during fermentation to reduce off-flavor by either directly denaturing the responsible enzymes, such as lipoxygenase, or degrading the compounds of undesirable aldehydes they produce.

#### 3.1.2. Precursor development

Cooked meat taste and aroma compounds are produced by complex chemical reactions. These include the Maillard reaction, oxidation of fatty acids, and thermal degradation of amino acids (Parliment, 1989; Shabbir et al., 2015). High-quality real meat contains higher concentrations of reducing sugars, fatty acids, and amino acids that are precursors to developing desirable meaty flavors during cooking (Piao et al., 2019; Xu et al., 2019; Stødkilde et al., 2021). In contrast, plant-based meat ingredients mainly contain complex proteins, polysaccharides, and oils, but not these key intermediate compounds (Li and Li, 2020). According to the current manufacturing procedure for plant-based meat analogs, hydrolysis protein sources and additional Maillard reaction substrates must be added to produce flavors comparable to those of real meat (Kale et al., 2022). It may be more efficient to degrade these macronutrients to promote the creation of meaty flavor intermediates without the use of flavorings by fermentation.

For the plant proteins used in alternative meats, including soy protein, pea protein, rice protein, and potato protein, several microorganisms showed high protease activity such as *B. subtilis*, *B*. *polyfermenticus*, and *B. amyloliquefaciens*, which produce large amounts of peptides and amino acids in fermented products (Sarkar and Tamang, 1995; Owens et al., 1997; Shrestha and Noomhorm, 2001; Cho et al., 2003; Shrestha et al., 2010; Jiang et al., 2019; Yang et al., 2019; Wang et al., 2021). Wang et al. (2021) measured water-soluble protein (WSP) in fermented soybean and compared it to non-fermented samples, as an indicator of the degree of protein degradation. Maximum protease activity of 694 U/g was correlated with 2.2-fold increases in WSP in the fermented soybean meal after a 48-h spontaneous fermentation process that subsequently decreased after 96 h. The study identified the predominate isolates as *B. amyloliquefaciens*, followed by other isolates such as LAB and *Pseudomonas*. The authors verified the positive impact of *B. amyloliquefaciens* on protein degradation by conducting a pure inoculation process into sterilized soybean meal. The study highlighted the importance of controlling several factors to improve the fermentation process and considered the water content of the medium as a key to either the success or failure of the fermentation process. A similar finding was reported by Yang et al. (2019) showing an increase of the degraded peptides by the protease activities of *B. amyloliquefaciens*. The authors found the degraded peptides ranging in size from 1 to 5 kDa (known as Maillard peptides) gradually increased from 20% to 72% at initial, but decreased after 60 h. Yeast species such as *Saccharomyces*, *Zygosaccharomyces*, *Kluyveromyces*, *Hansenula*, *Candida*, *Debaryomyces*, *Pichia*, and *Rhodoturola* (Lee et al., 1997; Arneborg et al., 2000; Deak, 2007; Rashad et al., 2011), filamentous fungi such as *Mucor flavus* (Cheng et al., 2009), *Aspergillus oryzae* (Kitamoto, 2002; Ohata et al., 2007), *Rhizopus oligosporus* and *Rhizopus oryzae* (Baumann and Bisping, 1995; Sparringa and Owens, 1999) were also reported to have high proteolytic activities. The resulting fermented products contain high concentrations of free amino acids (Dierick et al., 1974). Each of these microbes developed a unique amino acid profile correlated with identical sensory properties (DeMasi et al., 1990). During the fermentation of these products, several protease enzymes were found, including neutral and alkaline proteases with an optimal pH of approximately 7.0 and 10, respectively, which varied between species in various studies (Rao et al., 1998). For example, neutral proteases were reported with *B. subtilis* as well as fungal species such as *A. oryzae* (Chantawannakul et al., 2002; Sandhya et al., 2005). Other studies showed that *B. subtilis*, *B. licheniformis,* and *Aspergillus* sp. produced alkaline proteases (Çalık et al., 2000; Muthulakshmi et al., 2011).

Adding lipolytic microorganisms can also increase free fatty acid levels in the plant ingredients used in meat alternatives that contain oil such as soybean oil, canola oil, coconut oil, and sunflower oil. A wide range of microorganisms showed high lipolytic activity including species belonging to *Bacillus*, *Micrococcus*, and *Staphylococcus* (Cantoni et al., 1967; Debevere et al., 1976; Zahari et al., 2020), and lactic acid bacteria (LAB) such as *Lactobacillus casei*, *Lactcaseibacillus paracasei*, *L. plantarum*, and *P. acidilactici* (Liu and Hsieh, 2008). Yeasts such as *Yarrowia lipolytic* and *Candida boidinii* (Rodríguez-Gómez et al., 2010) and molds like *Penicillium* sp. (Rosa et al., 2009), and *R. oryzae* also produce lipases (Romano et al., 2007; Griebeler et al., 2011). Lipases can hydrolyse long-chain triglycerides to fatty acids, glycerol, and other intermediate compounds (Chandra et al., 2020). Besides, they also display esterification, and alcoholysis, which create a wide range of secondary metabolites, including ester compounds (Ergan et al., 1990; Chandra et al., 2020). Both fungal and bacterial lipases were found to be controlled by several factors, mainly pH, and temperature (Gunasekaran and Das, 2005). For example, *R. oryzae*, and *Mucor* sp. were reported to produce high lipase activities at 35°C and pH7 (Hiol et al., 2000; Abbas et al., 2002), *Penicillium* sp. at pH 5.5 and 47°C (Wolski et al., 2009), while *Bacillus* sp. at 50°C and pH 8 (Sharma et al., 2002). Other significant plant constituents used in meat substitutes are polysaccharides such as cellulose, pectin, and starch, where enzymes including cellulase, pectinase, and amylase have the potential capability to degrade them, respectively. Filamentous fungi are widely reported as potential cellulase producers such as *Trichoderma reesei* and *Aspergillus niger* (Dashtban et al., 2009). Endoglucanases and exogluconases make up the majority of the microbial cellulase family, which includes β-glucosidase and cellobiohydrolases, that break cellulose’s β-1,4 linkages into simple sugars (Jayasekara and Ratnayake, 2019). These enzymes can be found free or attached to the cell surface. Similarly, pectinolytic microorganisms including *Lactobacillus brevis*, *Erwinia herbicola*, *B. subtilis*, *Kluyveromyces fragilis*, and *S. cerevisiae* were shown to successfully degrade pectin (Agate and Bhat, 1966; Avallone et al., 2002). For starch breakdown, amylolytic microorganisms such as *A. oryzae*, *A. niger*, *B. subtilis*, *B. amyloliquefaciens*, and *B. licheniformis* can be used (Nigam and Singh, 1995; Owens et al., 2015). These enzyme activities were also associated with a decrease in the levels of polysaccharides and an increase in the concentration of reducing sugars (Ozyilmaz and Gunay, 2023).

The presence of these degraded compounds among plant-based meat ingredients produced through fermentation may increase the formation of desirable tastes and aromas during subsequent processing (cooking), improving the sensory quality to match that of meat products (Samelis et al., 1993; Johansson et al., 1994; Galgano et al., 2003; Zhang et al., 2010). Degraded compounds, including reducing sugars, free amino acids, and free fatty acids, can stand as precursors and interact with each other and develop various desirable meaty flavors such as alcohol, alkenes, ketones, aldehydes, ethers, esters, sulfur-containing compounds, and carboxylic acids, on the application of heat (Lorenzo, 2014). For example, a Maillard reaction can occur between free amino acids and reducing sugars during heat application producing various compounds, including 2-methyl-3-furanthiol, 4-mercapto-5-methyl-3(4H)-furanone, 2-methyl-3-methylthiofuran, and 2-methyl-3-methyldithiofuran that considered key compounds responsible for roast and meaty flavor (Liu et al., 2015). Additionally, straight-chain aldehydes, alcohols, and ketones derived from lipids have been found in high levels and shown to play a vital role in high-quality cooked meat (Elmore and Mottram, 2006). Furthermore, mono and di-unsaturated aldehydes showed to react with amino acids, mainly cysteine, and reducing sugar, ribose, to create 2-alkyl-(2H)-thiapyrans, and 2-alkyl-3-formylthiophenes, respectively, essential meat flavors (Campo et al., 2003; Elmore and Mottram, 2006). These findings showed the capacity of various microorganisms to convert complex plant materials components of protein, fat, and polysaccharides into more simple and suitable compounds such as free amino acids, free fatty acids, and reducing sugars which are considered crucial precursors to develop desired meaty flavor compounds when applying heat in plant-based meat analogs. These contributions are controlled by several factors including microbial species, and cultivation parameters, mainly time, temperature, pH, and water content.

#### 3.1.3. Creating generally desired volatiles

The resulting degraded molecules may also undergo further hydrolysis throughout the fermentation, yielding useful metabolites. For instance, certain bacteria can convert branched-chain amino acids (valine, leucine, isoleucine) or phenylalanine into volatiles like higher alcohols, aldehydes, and organic acids (Barbieri et al., 1992; Montel et al., 1998). Faba bean protein concentrate was fermented by *Weissella confusa* and about 40 various volatile compounds were identified (Tuccillo et al., 2022). The authors inoculated the protein concentrate with different initial inoculum ratios (10^5^–10^7^ CFU/g) under solid-state fermentation with the presence of sucrose, and the volatiles was detected by headspace solid-phase microextraction gas-chromatography mass-spectrometry. The resulting products performed at 30°C for 24 h were characterized by desirable ester compounds (fruity), as well as degrading the off-flavor aldehydes, resulting in potential improvement in the final product. Additionally, longer fermentation at higher temperature was correlated with higher levels of various metabolites, including ethyl acetate, ethanol, ethyl lactate, 2-methylfuran, and organic acids. The study also illustrated the non-significant effects of the inoculum ratio on the types of volatiles. Similarly, a variety of microbial metabolites such as acetaldehyde, diacetyl, 2, 3-butanediol, ethyl acetate, and ethyl butanoate that have desired flavor notes (fruity, buttery, floral) were created by different microorganisms, including other LAB, *Bacillus* spp., yeasts and filamentous fungi (Kanno and Takamatsu, 1987; Barbieri et al., 1992; Montel et al., 1998; Bramorski et al., 1998a,b; Sarkar and Nout, 2014; García-Cano et al., 2019; Elhalis et al., 2020, 2021). These substances are essential volatiles in real meat, and their inclusion in meat substitutes may improve the quality of the final product (Hoa et al., 2021). The metabolic pathways of such metabolites are well documented. For example, sugar and branched-chain amino acids can be metabolized by yeast, *S. cerevisiae*, and produce higher alcohols through anabolic reactions and multistep catabolic reactions, respectively (Ozyilmaz and Gunay, 2023). Similarly, esters are generated from the condensation of an alcohol and coenzyme-A-activated acid by alcohol acetyltransferase enzyme activities (Lambrechts and Pretorius, 2000). The concentrations and natural states of these microbial volatiles depend on several factors, including temperature, available nutrients, and microbial strains (Lambrechts and Pretorius, 2000).

It is worth mentioning that fermented real meat products, including sausage and cured meats showed several microbial metabolites that are linked to their characteristic desirable sensory quality. The presence of such metabolites might also be essential in plant-based fermented meat alternatives, to take plant-based meat to the next level. Microorganisms found in fermented meats, like sausages, have been linked to several functions. For instance, LAB including *Lactobacillus*, *Leuconostoc*, *Pediococcus*, *Lactococcus*, and *Enterococcus* produce organic acids from glucose by anaerobic glycolysis that lowers the pH (Bintsis, 2018). They also biosynthesis desirable volatiles including ketones, organic acids, aldehydes, and alcohols known for their characteristic flavor notes via various metabolic pathways. For example, during meat fermentation, ketones such as ethyl esters are produced by LAB, i.e., *Staphylococcus carnosus*, or by ethanol metabolization by mold, including *Penicillium nalgiovense* and *P. gladioli*, giving the fermented meat mushrooms and fruits aroma (Incze, 2004; Sharma et al., 2020). These microbial metabolites are linked to enhancing fermented meat safety, stability, and sensory quality (Laranjo et al., 2019). By applying fermentation to plant-based meat substitutes, the aroma profile of fermented meat may be replicated, resulting in improved fermented meat substitutes. To the best of the authors’ knowledge, this fermentation information is not available for plant-based meats but may be required as the next iteration step to bring plant-based meat to the next level. The use of plant-based fermented alternatives is not yet widespread on the market. Understanding the kinetic growth of the microorganisms and their metabolic activities might help design suitable fermentation processes to produce desirable flavor metabolites that enhance plant-based meat analogs, including fermented meat alternatives.

#### 3.1.4. Synthesis of key meaty volatiles

Other volatiles such as pyrazines and furans are key compounds present in cooked meat. These compounds are formed by the Maillard reaction and Strecker degradation which contribute to the roasted flavors of cooked meats (Hwang et al., 1994; Mottram, 1998; Rajini et al., 2011). Several fungi and bacteria species produce pyrazines, but little is known about the biogenesis processes involved (Rajini et al., 2011). For example, significant amounts of 2,5-dimethyl pyrazine, 3,6-dimethyl pyrazine, trimethyl pyrazine, and tetramethyl pyrazine were detected in natto (fermented soybean product) and were believed to be created by *B. subtilis* metabolic activities during fermentation (Kosuge et al., 1962; Leejeerajumnean et al., 2001). Pyrazine synthesis was found to occur by the end of the fermentation period, reaching about 80 mg/L after 6 days, which was associated with *Bacillus* cells hydrolysis and medium alkalization (Larroche et al., 1999). These processes were significantly impacted by the presence of various precursors in the medium and aeration rate. Maximum productivity was found at a medium aeration rate of about 0.005 volume of air sparged per unit volume of growth medium per minute (VVM), and with the presence of L-threonine and acetoin separately or sequentially at concentrations of 40 and 60 g/L, respectively. Related studies detected other pyrazine compounds associated with the microbial activities of *Aspergillus* species such as *A. flavus*, *A*. *sclerotorium* (Micetich and MacDonald, 1965), *Citrobacter freundii*, *Enterobacter agglomerans* (Robacker and Bartelt, 1997; Robacker and Lauzon, 2002), and *Corynebacterium glutamicum* (Demain et al., 1967). Similarly, furan derivatives can be produced by a wide range of microorganisms including *Bacillus coagulans*, *S. cerevisiae*, *Meyerozyma guilliermondii*, Kluyveromyces marxianus, and *Pichia stipites* (Domínguez de María and Guajardo, 2017; Gómez Millán and Sixta, 2020; Lalanne et al., 2021). For example, *K*. *marxianus* converted furan compounds including furfural and 5-hydroxymethylfurfural into corresponding alcohols such as furfuryl alcohol and 2,5-bis(hydroxymethyl)furan, respectively, under anaerobic fermentation by NADPH-dependent aldehyde reductase, KmGRE2, while aerobic conditions produced organic acids such as furoic acids (Akita et al., 2015; Baptista et al., 2021). Another group associated with meaty aroma formation is sulfur-containing amino acids that play an essential role in the production of key meaty flavors through thermal degradation or reaction with reducing sugars. Some plant proteins like those from legumes have suboptimal levels of sulfur-containing amino acids, thus, adding such compounds is essential in the current plant-based meat analogs production process (Krishnan and Jez, 2018). Microbial biosynthesis of these key amino acids can be achieved using bacteria such as *Bacillus vallismortis*, and *B. subtilis*, which produced 56.3 and 42.8 mg/L of cysteine in sorghum extract, respectively (Shen et al., 2020). Several microorganisms isolated from dairy products, plants, and grains were shown to produce thiamine (LeBlanc et al., 2017). For example, *Lactobacillus sanfranciscensis* isolated from traditional sourdough (Vogel et al., 2011), *Weissella koreensis* from kimchi (fermented vegetable; Jeong et al., 2018), as well as *Neurospora crassa* and *A. oryzae* were shown to form thiamine at different quantities (Kubodera et al., 2003; Cheah et al., 2007). The presence of sulfur sources, including inorganic and organic, is essential to the biosynthesis of microbial sulfur-containing amino acids (Thomas and Surdin-Kerjan, 1997). For example, in yeast, sulfate is firstly phosphorylated to phosphoadenylyl sulfate which is reduced to sulfite then sulfide by sulfite reductase that is subsequently incorporated into carbon chains to create cysteine and methionine (Thomas and Surdin-Kerjan, 1997). The inclusion of such microbial metabolites in plant-based meat analogs might improve final product quality by replicating meaty tastes and aromas. These findings demonstrate the ability of the selected microorganisms to synthesize desired key aroma compounds found in real meat, including pyrazines, furans, and sulfur-containing amnio acids that are known to be present in high-quality meat products. Selecting the right microorganism and optimizing the incubation parameters, mainly the nutrient sources in the medium, are essential to the biosynthesis of such metabolites. Future research comparing the quantities of these microbial volatile compounds to those in meat products could aid the development of a suitable strategy to mimic the volatile profile of meat products via fermentation.

#### 3.1.5. Flavor challenges

Excessive fermentation may lead to the accumulation of aroma compounds, such as esters, that may form an overly fruity aroma in plant-based meat analogs, which may be undesirable. For example, concentrations of ethyl acetate and isoamyl acetate were 4-fold higher with the presence of high sugar content during *S. cerevisiae* brewer’s wort fermentation causing undesirable overlay fruity aroma (Hirst and Richter, 2016). Younis and Stewart (1999) reported a significant reduction of these fruity aroma compounds by supplementing the Worts with maltose syrups and achieved 10% and 40% of ethyl acetate and isoamyl acetate, respectively. Such knowledge might be applied to plant-based meat analogs. Uncontrolled fermentation also may lead to producing other off-flavor compounds such as sulfur-containing compounds, propionic acid, ferulic acid, and 2,3-pentanedione that may negatively impact the organoleptic quality of the fermented products (Liu, 2015). Sulfides, polysulfides, thiols, thioesters, and heterocyclic compounds are examples of aromatic sulfur compounds that cause rotten eggs, cabbage, and onion aromas with a very low sensory threshold in foods, including wine by *S. cerevisiae* (Swiegers and Pretorius, 2007). Hydrogen sulfide, for example, was found to be created under cysteine and methionine-limited conditions, resulting in excessive sulfide that is converted to H_2_S (Thomas and Surdin-Kerjan, 1997). Excessive enzymatic activities might also create an undesirable breakdown of peptides, amino acids, and fatty acids, as well as converting nitrogen compounds into ammonia that are characterized by an undesirable pungent and fishy odor (Martínez-Álvarez et al., 2017). *Aspergillus* species, such as *A*. *sydowii*, were shown to produce excessive amounts of extracellular endo- and exo-peptidase enzymes that were correlated to an undesirable rancid odor during cheese ripening processes (Takenaka et al., 2021). The study highlighted the essential presence of antioxidant enzymes such as catalase B and Cu/Zn superoxide dismutase produced by *Aspergillus* species, including *A. chevalieri* and *A*. *pseudoglaucus*, that minimized the oxidative damage of lipids, preventing fishy flavor production during the ripening process (Takenaka et al., 2021). Similarly, alkaline *Bacillus* fermentation is associated with the creation of excessive amounts of ammonia, and sometimes a polypeptide of glutamic acid, giving the final product a notably stringy and pungent smell, which may be undesirable in plant-based meat analogs (Escamilla et al., 2019; Elhalis et al., 2023a). In both cases overly fruity and off-flavors might negatively impact the purpose of creating meat analogs and should be considered.

### 3.2. Texture and mouthfeel

As mentioned in previous sections, current texturization technologies face several limitations in the creation of meat-like fibrous structures and appearance. These limitations may be circumvented via the use of microorganisms and fermentation technology.

#### 3.2.1. Bacteria

It has been demonstrated that introducing a *B. subtilis* fermentation step during the manufacturing of meat analogs results in products with desirable textural properties, such as increased chewiness, integrity, and hardness, compared to non-fermented products (Gu et al., 2020). The authors inoculated texturized protein, isolated soy protein, wheat gluten, and corn starch with *B*. *subtilis* (5% w/w), and it was kept at 37°C for 60 h with a 50% moisture content. The physical changes, including hardness, springiness, cohesiveness, and chewiness were measured by compressing the sample with a cylinder probe. The study showed that crucial reduction, mainly in hardness and chewiness, occurred in the fermented samples that were potentially affected by fermentation time. The authors correlate these changes to the *B. subtilis* enzymatic activities of esterase, neutral proteases, and alkaline proteases that alter the protein structure. Such a conclusion was further confirmed by scanning electron microscopy, which showed a gradual disappearance of air cells, a diminished protein network, and the formation of more layered structures during fermentation (Gu et al., 2020). Similar studies illustrated the change in texture properties could be linked to microbial proteolytic activities. Proteolysis increased the number of exposed non-polar groups, which increased the hydrophobicity of the fermented mass. Such changes correspond to a significant increase in the water and oil-holding capacities, which is associated with improved juiciness, reduced chewiness, and hardness of the products resemble cooked meat (Periago et al., 1998; Akubor and Chukwu, 1999; Chandra-Hioe et al., 2016; Oloyede et al., 2016; Çabuk et al., 2018; Razavizadeh et al., 2021). Adding hydrocolloids to protein-based raw materials is another method to replicate the fibrous structure of both fresh and cooked meat (Dekkers et al., 2018a; Razavizadeh et al., 2021). Agricultural side-streams that contain hydrocolloids such as husk and okara are considered waste and used mainly for feeding animals due to their unpleasant sensory quality, low digestibility, and inclusion of anti-nutritional factors (Ancuța and Sonia, 2020). LAB fermentation using *L. plantarum*, and *L. acidophilus* was reported to improve the digestibility, sensory quality, and fibrous structure of these side streams, making them suitable to be used in meat analogs (Ferri et al., 2016; Behera et al., 2018; Šertović et al., 2019; Razavizadeh et al., 2021). Generally, fermentation may be used to modulate the texture of the main ingredients, mostly through microbial enzymatic activities that might change their internal structure, including fiber and protein components, as well as fermenting hydrocolloids and improving their sensory quality by suitable microorganisms and using them as fibrous additives. These approaches might be used to improve the production of plant-based meat alternatives by choosing the appropriate microorganisms and optimizing fermentation parameters, especially ingredients and time.

#### 3.2.2. Fungi

Filamentous fungi fermentation produces biomass that has a texture and structure similar to the muscle fibers of real meat (Han et al., 2005; Asgar et al., 2010). Recent attention has been given to filamentous fungi due to their high protein concentration, complete amino acid profile, low-fat content, high digestibility, and high fiber content (Kinsella and Franzen, 1978; Ajwalia, 2020). Edible filamentous fungal strains, including *A. oryzae*, *R. oryzae*, *Fusarium venenatum*, and *Neurospora intermedia* showed efficient growth and improved the functional and nutritional properties of various fermented products (Williamson et al., 2006; Ruxton and McMillan, 2010; Day, 2013; McCarthy et al., 2016; Souza Filho et al., 2018; Upcraft et al., 2021). Such filamentous species contain high-quality protein, and their mycelium is rich in polyunsaturated fatty acids and fibers that can be converted to a meat-like texture using a controlled denaturation process (Finnigan et al., 2017; Kumar et al., 2017). Quorn, for instance, is considered one of the most successful mycoproteins in the current market that are produced by filamentous fungi, *F*. *venenatum*. *F*. *venenatum* is a single-cell protein that can proliferate and form biomass. This biomass is transformed into meat analog products by adding flavorings and undergoing texturizing processes (Wood and Tavan, 2022). Fungal mycelium is composed of natural polymers of cellulose, chitin, and proteins, and is considered a natural fibrous material. The basic structure of fungal mycelium is called hyphae, which are formed from fungal spore germination (Iram et al., 2022). Several factors were listed as affecting the fungal hyphal growth and their morphology. The diameter, shape, length, and arrangement of hyphae vary between species (Iram et al., 2022). Agitation was shown to negatively impact the hyphae growth and length by either causing pellet breakdown or shaving the hyphae extension (Lejeune, 1996; Cui et al., 1997). The medicinal fungi *Ganoderma lucidum and Pleurotus ostreatus* were cultivated on cellulose and potato-dextrose substrates, and their fibrous structure formation was observed (Haneef et al., 2017). The study illustrated various factors that have an impact on hyphal morphology, including time, species, and growing substrates. For example, *P*. *ostreatus’* hyphae were larger in diameter compared to *G. lucidum*, regardless of the feeding substrates. The density of hyphae increased with the growth. A scanning electron microscope (SEM) showed *G. lucidum* had a short and tube-like entangled structure at the growth beginning, which converted into a more compact filamentous structure with time, while *P*. *ostreatus* hyphae showed one unique kind of compressed filamentous throughout the whole growth phase. Similarly, chemical analysis showed *G. lucidum* mycelium contained more lipids while *P*. *ostreatus* had more polysaccharides. These chemical and morphological changes were correlated to various hydrodynamic, thermomechanical, and mechanical characteristics of both types of hyphae. Regardless of the difference between filamentous fungi and medicinal fungi, such knowledge may be valuable when researching filamentous fungi, finding factors that impact their mycelium characteristics, and applying them to plant-based meat analogs. Knowledge about filamentous fungal species, their ability to ferment plant ingredients, and the physiochemical characteristics of their fibrous mycelium materials might be a possible approach to improving the plant-based meat analogs’ fibrous texture, but there is still a lot of room for improvement and further research is required.

#### 3.2.3. Texture challenges

Microbial enzymes and the acidification process that occur frequently during fermentation may be associated with significant changes in product texture, which needs to be investigated further for their suitability in plant-based alternative meat. The acidification process that can occur during fermentation might change the protein properties of the fermented sample. Lactic acid produced by LAB caused a decrease in the casein surface negative charge to close to neutral as the isoelectric point approached, resulting in casein aggregation (Lucey et al., 2000). This denaturation process was associated with significant changes in the texture of the product, including gelation properties (Lucey, 2004). Similarly with starch, although its concentration in plant-based meat analogs is low for example, it is worth mentioning the adverse impact that might be occurred during fermentation. For example, when cassava starch fermented with *B*. *subtills, Lactobacillus casei*, *L. plantarum*, and *Candida krusei*, demonstrated lower water uptake, less swelling capability, low adhesiveness, low viscosity, and undesirable gelation when compared to non-fermented samples (Numfor et al., 1995). Such characteristics were believed to be linked to higher microbial enzymatic activities, such as amylases, which increased amylose content and caused changes in the internal granular structure of fermented cassava starch (Numfor et al., 1995; Harper et al., 2022). Another example of the negative impact of overfermentation is kimchi (fermented vegetables), which lost firmness, chewiness, and crispy texture characteristics and became softer (Cheigh et al., 1994). The enzymatic activities of several microorganisms isolated during kimchi fermentation, including *B. subtilis*, *Candida pseudotropicalis*, *Saccharomyces fragilis*, *Oospora lactis*, *Penicillium* spp., and *Aspergillus* spp., were mainly responsible for the softening. These isolates were abundant at the end of fermentation and produced a variety of enzymes, including pectinases, which degrade vegetable tissue structures, including pectic substances (Cheigh et al., 1994; Surh et al., 2008). Uncontrolled and excessive fermentation of plant ingredient components such as starch and protein can result in undesirable alterations and poor texturized ingredients such as softening and aggregation. Such unfavorable effects must be recognized and regularly evaluated for fermentation to be effectively implemented into plant-based meat analogs.

### 3.3. Appearance

Fresh real meat possesses a bright red color that changes to brown after cooking. Hence, it is preferred that meat analogs have the same color attributes (Ishaq et al., 2022). Several synthetic dyes were found to cause health problems (i.e., carcinogenic) and were banned by the Food and Drug Administration (FDA), causing a rise in global demand for natural pigments (Manikprabhu and Lingappa, 2013; Sajjad et al., 2020). Pigments that originate from plants were reported to have poor stability when exposed to excess heat, light, and inappropriate pH. These pigments may also have low water solubility and may not be available throughout the year, which limits their applications in processed foods. On the other hand, several microbial pigments were applied as food colorants and reported to possess other benefits including antimicrobial, antioxidative, and anticancer activities. Microbial pigments such as astaxanthin, β-carotene, violacein, lycopene, prodigiosin, canthaxanthin, and riboflavin have been successfully used in various food industries, including soft drinks, cooked sausages, fish, candy, cheese, fruits, meat, snacks, beverages, wine, beer, and baked items (Dharmaraj et al., 2009; Namazkar and Ahmad, 2013; Konuray and Erginkaya, 2015; Dufossé, 2018). These microbial pigments might be suitable to be used as alternatives to enhance the appearance of plant-based meat analogs (Sajjad et al., 2020).

#### 3.3.1. Fungi

Several fungi, including *Y*. *lipolytica*, *Aspergillus*, *Monascus*, *Fusarium*, *Penicillum*, *Neospoare*, and *Trichoderma* produced various pigments as secondary metabolites during their growth (Mukherjee et al., 2017). Monascus *purpureus* and *Aspergillus nidulans* were reported to produce red pigmentation in several studies and might be suitable to be applied to plant-based fresh meat alternatives (Francis, 1996; Stchigel et al., 1999; Babitha et al., 2008). According to a related study, maximum fungal biomass and extracellular red pigment were produced by *M. purpureus* when *M. purpureus* was grown in complete darkness. The study also illustrated the potential production of various pigments by other fungal species such as *A. nidulans*, *Fusarium verticillioides*, and *Penicillium purpurogenum* (Velmurugan et al., 2010). Similar findings were recently reported by using *Rhodotorula mucilaginosa* and *M. purpureus* to efficiently produce fresh meat-like pigments that can be successfully applied in plant-based meat alternatives (Ou et al., 2023). The authors inoculated soybean proteins and studied the impact of various parameters, such as inoculum size, moisture content, and temperature, on the resulting color changes. The study showed that the value of redness, as measured by chromatic aberration, increased with fermentation time, with the fastest increase between 8 and 12 h and optimum color formation at 30°C for 24 h. The optimum inoculation rates for *M. purpureus* and *R. mucilaginosa* were about 1.5% and 3%–5%, respectively. Moreover, coculturing *L. plantarum*, promoted the growth of the main fermenting microorganisms, improved the resultant protein appearance, and enhanced the fermented protein sensory quality by significantly increasing the levels of total furan, and ester compounds. *Neurospora* species can also be used to produce red pigments. This fungal species has high protease, amylase, and phytase activities. *N. intermedia* creates yellow to orange pigments and has been used in the production of traditional Indonesian food (known as Oncom Merah) to increase the food’s microbial safety (Perkins and Davis, 2000; Nout and Aidoo, 2011). Furthermore, *N. intermedia* biomass is rich in essential amino acids and fatty acids, making it a promising fermentation microorganism to be used in plant-based meat alternatives (Pandit and Maheshwari, 1996; Ferreira et al., 2014). Several factors were reported to affect the fungal biomass growth of *N. intermedia* and their pigment production, including carbon, nitrogen, light, pH, and minerals. Among them, low pH and high aeration were found to trigger the biosynthesis process for pigment synthesis (Gmoser et al., 2018). Similarly, *N. crassa* improved the nutritional quality, and digestibility, and decreased allergenicity when used to ferment soybeans under a solid-state process (Li et al., 2019). These characteristics demonstrate the abilities of *Neurospora* species, leading the authors to hypothesize that they would be suitable for use as the primary fermentation microbe or in a coculture process to produce safer and more nutritious plant-based meat substitutes. The brown pigment was also observed with *Yarrowia lipolytica* in the presence of tyrosine in cheese agar (Gardini et al., 2006). *Y*. *lipolytica* was reported to have high proteolytic and lipolytic activities and was able to oxidize tyrosine to melanin (brown color), catalyzed by tyrosinase (Carreira et al., 1998). Browning of cooked meat may also be produced indirectly by microbial activity. As previously stated, several microbial enzymes are responsible for the breakdown of plant protein and polysaccharide components, which in turn form brown color (melanoidins) during cooking via the Maillard reaction, besides forming the aroma compounds as mentioned under the flavor discussion. Melanoidins are water-soluble polymers formed in the late stage of the Maillard reaction via various reactions, including polymerization, cyclization, and degradation (Wang et al., 2011). Browning pigments produced through the Maillard reaction were linked to brown color change in soy sauce, roasted coffee, bread crust, and cooked meat (Murata, 2021).

#### 3.3.2. Microalgae

Algae such as *Haematococcus pluvialis* are an important source of natural red pigment in large-scale production (astaxanthin), in addition to its high protein level (Hosseini et al., 2020). Xia et al. (2022) mixed the *H. pluvialis* residue with pea protein and evaluated its contribution to the color and texture of the resulting meat analogs. The outcome showed potential changes in the meat alternative’s appearance that mostly mimics natural red real meat and improvements in the product’s fibrous structure. The mechanism of astaxanthin accumulation in microalgae cells is not fully clear, but the presence of sucrose at a concentration of 1–10 mM in the medium under high light stress is associated with about a 30% increase in the pigment yield of *H. pluvialis* (Du et al., 2021). Related studies showed the capability of several microalgae strains to be cultivated and grow on various agricultural residues including soybeans, peas, and corn seeds (Shirai et al., 1998; Koutra et al., 2018; Stiles et al., 2018; Chong et al., 2021). Additionally, the development of consortia of microalgae with bacteria or fungi species has recently widened microalgae applications (Renuka et al., 2018). These results support the authors’ hypothesis that microalgae could be used as an ingredient in meat analogs (as a source of protein or colorants) or combined with other microorganisms (consortia) during fermentation of plant-based raw ingredients to alter the appearance and texture of the final product.

#### 3.3.3. Bacteria

Food-grade pigments isolated from bacterial species were reported to have colorant stability in addition to their preservative and antioxidant properties (Nigam and Luke, 2016). Carotenoid colors ranging from yellow to dark red and brown were delivered by several bacteria, for example, *Serratia marcescens*, *Serratia rubidaea*, *Xanthophyllomyces dendrorhous*, *Agrobacterium aurantiacum*, *Bacillus* sp., *Bradyrhizobium* sp., and *Carotinifaciens paracoccus* (Malik et al., 2012; Mohana et al., 2013; Kirti et al., 2014). These bacterial species can be cultured in agriculture by-products like sugarcane molasses, beet molasses, cheese whey, peat hydrolysates, and glucose syrup (Velmurugan et al., 2010). This ensures that the production of these pigments is both cost-effective and friendly to the environment. To the best of the authors’ knowledge, no attention has been made to using bacterial strains to modify plant ingredient color.

#### 3.3.4. Color challenges

Some microalgae species have several undesirable characteristics that may prevent their use in meat analogs. Various species of *diatoms*, *cyanobacteria*, and *dinoflagellates* produce toxins, and only a few commercial strains have been developed. These commercial strains are safe, but their production cost is high (Chen et al., 2012; Helmy et al., 2023). Currently, a few strains such as *Chlorella vulgaris*, *Spirulina platensis*, *Haematococcus pluvialis,* and *Dunaliella salina* are used in high-value food applications like health supplements or the production of bioactive compounds as it is costly to grow and maintain these microalgae strains at scale (Borowitzka, 2013). Some microalgae, like *S. platensis*, were also found to be allergenic (Lafarga, 2019). Microalgae can also be difficult to digest as they contain cellulosic cell walls (Tchorbanov and Bozhkova, 1988). Therefore, post-processing must be applied to enhance the digestibility of microalgae. This can be done by disrupting the cellulosic cell walls using hydrolytic enzymes, such as pancreatic enzymes. By doing so, it is possible to increase the digestibility of *C. vulgaris* and *S. platensis* by 70% and 97%, respectively (Kose et al., 2017). Finally, some microalgae can impart an unpleasant fishy odor and undesirable green color. These characteristics negatively impact the sensory characteristics of microalgae products, which limit the application of microalgae in food. These unpleasant sensory properties can be modulated by changing several factors, including altering the medium components, changing the harvest time, and applying post-processing steps like cooking (Milovanović et al., 2015; Isleten Hosoglu, 2018). Therefore, it is necessary to select suitable microalgae and include post-processing treatments to remove the undesirable characteristics of microalgae (Helmy et al., 2023). Microalgae were shown to produce pigments that mimic the red color of meat; however, persisting in the red color after cooking may also raise consumer concern. Overall, despite the presence of microbial pigments that may mimic the raw and cooked meat color, finding pigments that can change from red to brown is essential. To the best of the authors’ knowledge, no significant efforts have been devoted to identifying such pigments to be applied in plant-based meat analogs. These findings demonstrated that diverse microorganisms might be a potential source of natural red and brown pigments, as well as help in the creation of essential Maillard reaction precursors and the formation of brown pigments during heat treatment. These approaches can be applied to meat analogs and mimic fresh and cooked meat reducing the need for additives. Its favorable application is determined by the selected species as well as a variety of incubation parameters. Further research is required to screen for suitable candidates for food applications and optimize their manufacturing processes, considering the pigment’s stability, its cost, and sensory characteristics. Table 2 summarizes the desirable and undesirable sensory attributes of selected microorganisms.

**Table 2:** Microorganisms’ contributions to the sensory quality of plant-based meat.

Fermentation by	Contributions to	Description	References
Aspergillus flavus/Aspergillus sclerotorium/*Citrobacter freundii*/*Enterobacter agglomerans*/ *Corynebacterium glutamicum*	Flavor	Biosynthesize pyrazines	Micetich and MacDonald (1965), Demain et al. (1967), Robacker and Bartelt (1997), and Robacker and Lauzon (2002)
	Texture	Loss of firmness and chewiness if over fermented	Cheigh et al. (1994)
*Aspergillus niger/Bacillus licheniformis*	Flavor	Breaks down polysaccharides for flavor development	Nigam and Singh (1995) and Owens et al. (2015)
*Aspergillus oryzae*	Flavor	Breaks down proteins and polysaccharides for flavor development	Nigam and Singh (1995), Kitamoto (2002), Kubodera et al. (2003), Ohata et al. (2007), and Owens et al. (2015)
		Produces sulfur-containing amino acids (precursors to key meaty flavors)	
	Texture	Has similar structure and texture as meat	Asgar et al. (2010)
*Aspergillus nidulans/Fusarium verticillioides/Isaria farinose/Monascus purpureus/Neurospora crassa/Penicillium purpurogenum/Rhodotorula mucilaginosa*	Appearance	Produces red pigments	Francis (1996), Stchigel et al. (1999), Perkins and Davis (2000), Babitha et al. (2008), Velmurugan et al. (2010), Nout and Aidoo (2011), and Ou et al. (2023)
*Aspergillus species, such as A. sydowii*	Flavor	Excessive fermentation causes rancid odor	Takenaka et al. (2021)
*Bacillus amyloliquefaciens*	Flavor	Masks beany off notes	Cho et al. (2003), Jiang et al. (2018), Montel et al. (1998), Nigam and Singh (1995), and Wang et al. (1997)
		Denatures lipoxygenase	
		Produces desirable flavors	
		Produces sulfur-containing amino acids (precursors to key meaty flavors)	
		Breaks down proteins and polysaccharides for flavor development	
*Bacillus coagulans/Meyerozyma guilliermondii/ Pichia stipites*	Flavor	Biosynthesize furans	Domínguez de María and Guajardo (2017) and Gómez Millán and Sixta (2020)
*Bacillus polyfermenticus*	Flavor	Masks beany off notes	Shrestha et al. (2010) and Jiang et al. (2018)
		Denatures lipoxygenase	
		Breaks down proteins for flavor development	
*Bacillus subtilis*	Flavor	Masks beany off notes	Kosuge et al. (1962), Kanno and Takamatsu (1987), Nigam and Singh (1995), Owens et al. (1997), Wang et al. (1997), Leejeerajumnean et al. (2001), Jiang et al. (2018), and Shen et al. (2020)
		Denatures lipoxygenase	
		Produces desirable flavors	
		Produces sulfur-containing amino acids (precursors to key meaty flavors)	
		Breaks down proteins and polysaccharides for flavor development	
	Texture	Improves chewiness, integrity, and hardness	Gu et al. (2020)
	Flavor	Produces undesirable flavors (ammonia and bitterness)	Escamilla et al. (2019)
	Texture	Decreases water uptake, swelling, and viscosity	Cheigh et al. (1994), Numfor et al. (1995), and Elhalis et al. (2023a)
		Decreases firmness and chewiness if over fermented	
		Increases stickiness	
*Bacillus vallismortis/Lactobacillus sanfranciscensis/Weissella koreensis/Neurospora crassa*	Flavor	Produces sulfur-containing amino acids (precursors to key meaty flavors)	Cheah et al. (2007), Vogel et al. (2011), Jeong et al. (2018), and Shen et al. (2020)
*Candida*	Flavor	Breaks down proteins for flavor development	Lee et al. (1997)
	Texture	Loss of firmness and chewiness if over fermented	Cheigh et al. (1994)
*Debaryomyces/ Hansenula/Mucor flavus/Pichia/ Rhodoturola*	Flavor	Breaks down proteins for flavor development	Lee et al. (1997), Cheng et al. (2009), and Rashad et al. (2011)
*Fusarium venenatum*	Texture	Has similar structure and texture as meat	Finnigan et al. (2017) and Kumar et al. (2017)
*Haematococcus pluvialis*	Appearance	Produces red pigments (astaxanthin)	Renuka et al. (2018), Hosseini et al. (2020), and Xia et al. (2022)
	Texture	Improves the fibrous structure	Xia et al. (2022)
	Appearance	Red pigments may persist even after cooking	Yang et al. (2019)
*Kluyveromyces marxianus*	Flavor	Masks beany off notes	Rashad et al. (2011) and Hu et al. (2019)
		Denatures lipoxygenase	
		Breaks down proteins for flavor development	
		Produces desirable flavors	
*Lactobacillus acidophilus*	Texture	Improves fibrous structure	Šertović et al. (2019)
		Increases the water and oil-holding capacities	
	Texture	May cause undesired gelation	Lucey (2004)
*Lactobacillus casei*	Flavor	Breaks down fats for flavor development	Liu and Hsieh (2008) and García-Cano et al. (2019)
		Produces desirable flavors	
	Flavor	Produces undesirable flavors	Lucey (2004)
	Texture	May cause undesired gelation	Harper et al. (2022)
*Lacticaseibacillus paracasei*	Flavor	Breaks down fats for flavor development	Liu and Hsieh (2008) and García-Cano et al. (2019)
		Produces desirable flavors	
	Texture	May cause undesired gelation	Lucey (2004)
*Lactiplantibacillus plantarum*	Flavor	Breaks down fats for flavor development	Liu and Hsieh (2008) and García-Cano et al. (2019)
		Produces desirable flavors	
	Texture	Improves fibrous structure	Ferri et al. (2016) and Behera et al. (2018)
		Increases the water and oil-holding capacities	
	Flavor	Produces undesirable flavors	Lucey (2004)
	Texture	May decrease water uptake, swelling, adhesiveness and viscosity	Numfor et al. (1995) and Harper et al. (2022)
		May cause undesired gelation	
*Lindnera saturnus*	Flavor	Converts undesirable aldehydes to desirable ester compounds	Vong and Liu (2018)
*Neurospora intermedia*	Appearance	Produces red pigments	Perkins and Davis (2000) and Nout and Aidoo (2011)
	Texture	Has similar structure and texture as meat	Finnigan et al. (2017) and Kumar et al. (2017)
*Yarrowia lipolytica*	Appearance	Produces brown pigments	Gardini et al. (2006)
*Pediococcus acidilactici*	Flavor	Breaks down fats for flavor development	Liu and Hsieh (2008)
*Rhizopus oligosporus*	Flavor	Breaks down proteins and polysaccharides for flavor development	Baumann and Bisping (1995), Nigam and Singh (1995), Sparringa and Owens (1999), and Owens et al. (2015)
*Rhizopus oryzae*	Flavor	Breaks down proteins and fats for flavor development	Baumann and Bisping (1995), Bramorski et al. (1998a), Sparringa and Owens (1999), and Griebeler et al. (2011)
		Produces desirable flavors	
	Texture	Have similar structure and texture as meat	Asgar et al. (2010)
*Saccharomyces cerevisiae*	Flavor	Breaks down proteins for flavor development	Arneborg et al. (2000) and Domínguez de María and Guajardo (2017)
		Produces desirable flavors	
		Biosynthesize furans	
*S. cerevisiae*	Flavor	Causes overlay fruity aroma	Hirst and Richter (2016)
*S. cerevisiae*	Flavor	Forms Sulfides, polysulfides, thiols, thioesters, and heterocyclic cause rotten eggs, cabbage, and onion aromas	Swiegers and Pretorius (2007)
*Zygosaccharomyces*	Flavor	Breaks down proteins for flavor development	Arneborg et al. (2000), Domínguez de María and Guajardo (2017), Gómez Millán and Sixta (2020), and Lalanne et al. (2021)
		Produces desirable flavors	

## 4. Plant-based meat analogs and starter culture technology

**Figure 3:**
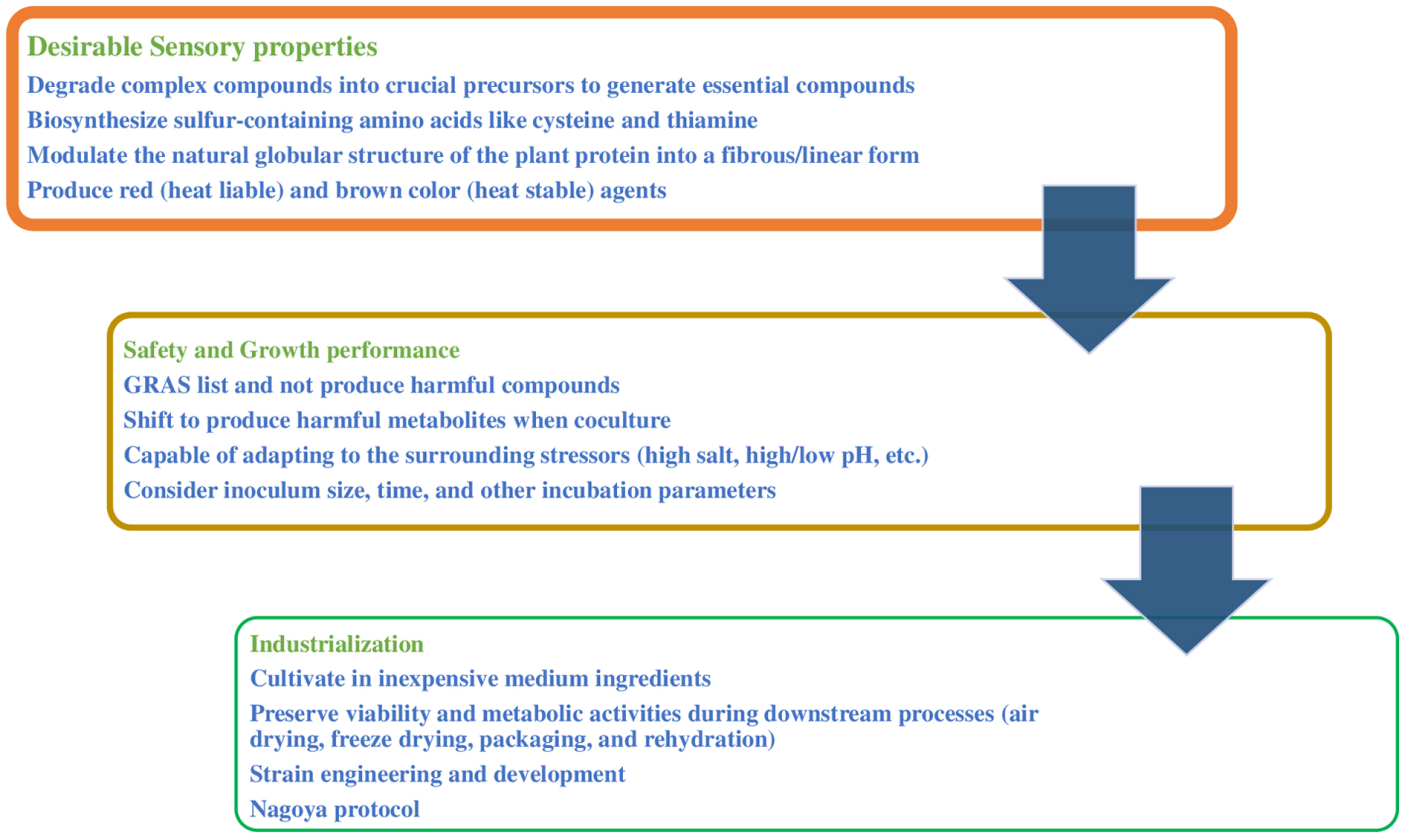
Starter culture considerations in plant-based meat analogs.

Fermentation was shown to improve the sensory quality of plant-based meat and starter cultures; however, further considerations are needed to be controlled. The conversion from back slopping (a small amount of fermented products is added into fresh ingredients) to modern microbial starter cultures has been shown the best way to control the fermentation process (Wirawati et al., 2019). A starter culture is live microbial preparation consisting of one or more microbial strains, including bacteria and fungi, to be added to raw ingredients and produce desirable fermented foods (Herody et al., 2010). Starter cultures have been used to improve the texture, flavor, appearance, and nutritional quality of tempeh, bread, cheese, yogurt, coffee, and sausage to satisfy consumer preferences (Steinkraus, 2004; Jia et al., 2021; Korcari et al., 2021; Zhou et al., 2021; Papaioannou et al., 2022; Elhalis et al., 2023a,b). So far, no starter cultures have been created to fulfill these characteristics. However, the success and widespread use of starter culture technology in different food products makes the authors believe in its application in plant-based meat alternatives. To select suitable starter cultures, it is important to assess the microorganism’s performance to conduct the desired biotransformation and ensure its safety and potential for commercial development (Figure 3). Initially, searching for suitable microbial candidates among the reported studies. Additionally, several modern bioinformatic tools such as the Kyoto Encyclopedia of Genes and Genomes (KEGG) database might also be applied to help scientists look for suitable microbial candidates (Pérez-Miranda et al., 2007; Helmy et al., 2020, 2023). Subsequently, the selected candidates are subjected to several laboratory assays to ensure their suitability to plant-based meat analogs, these include.

### 4.1. Sensory properties

Special attention should be given to strains that can modulate the amino acid profile, texture, and color of the ingredients. For instance, it is recommended to choose strains that can biosynthesize sulfur-containing amino acids like cysteine and thiamine as precursors to produce meat flavors when cooking. Microorganisms might produce aroma and sour compounds which are desirable in some plant-based meat analogs including fermented meat alternatives (Chandan, 2014; Gustaw et al., 2021). However, microorganisms that produce unsuitable aroma compounds in plant-based meat alternatives, including overly fruity aroma, and sour, bitter, or off flavors should be avoided.

Research should be done on the strains that have been reported to modulate the natural globular structure of the plant protein into a fibrous/linear form. Filamentous fungi mycelium attached to plant ingredients might help to mimic the texture of meat. Some microbial species also improved the hardness and biting quality of the final product including *Bacillus* as mentioned above. Similarly, it is valuable to study strains that naturally produce red (heat liable) and brown color (heat stable) agents that might be used together to mimic raw and cooked meat, respectively, considering pigments’ stability and their capability to convert from red to brown during cooking to mimic the meat color change.

Data showed that most of the raw materials lack important micro compounds, such as amino acids, fatty acids and reducing sugars, which are considered crucial precursors to generate essential compounds in the final products during heat treatment. On contrast, they contain complicated proteins, carbohydrates and lipids. Selecting starter cultures with strong capability to break down these complex compounds may be essential. Degradation of these substances creates low molecular weight substances that drastically alter the texture, flavor, and aroma, as mentioned above. Several traditional and advanced techniques have been used to measure such microbial activities, including plate assay, colorimetry, chromatography, and microcalorimetry (Thomson et al., 1999; Kasana et al., 2011; Li et al., 2021).

### 4.2. Safety and growth performance

When designing starter culture to be used in foods, their safety is a crucial aspect. The selected strain has to be among the generally recognized as safe (GRAS) list (Russo et al., 2017). Additionally, to consider coculture, further consideration that some strain shift to produce harmful metabolites, including mycotoxins and biogenic, in the presence of another microbial strain as well changing the incubation parameters. For example, co-culturing *Fusarium tricinctum* and *Fusarium begonia* induced to accumulate of subenniatins (cytotoxic compounds), which were not present when either of the two molds was cultivated alone (Meca et al., 2010; Wang et al., 2013). Similarly, higher levels of aflatoxin B1 were found when inoculating hydrated textured soy protein with *Aspergillus parasiticus* and *Penicillium citrinum* at 21°C than that at 31°C, and at 80% RH than that at 93% (Rao, 1985).

The fermentation performance of starter cultures is a key selection criterion for the successful fermentation process. Selected strains should be capable of adapting to the surrounding stressors, which are often present in fermentation mass such as high temperature, high salt, alcohols, and pH. This criterion can be determined by exposing the selected strains to various stressors and observing their behavior. Successful multiplication under such stressful conditions is considered an essential indicator of their high fermentation performance. Other factors such as inoculum size, inoculation time, and incubation parameters should be studied to optimize the fermentation process. Similar approaches were successfully applied to design starter cultures for other foods (Pereira et al., 2012; dos Santos Cruxen et al., 2019; Elhalis et al., 2021).

### 4.3. Industrialization

The chosen microorganism should be suitable for commercial applications, including its cultivability, viability, vitality, and storage stability. The suitability of the selected microorganism to grow in inexpensive medium ingredients during upstream cultivation, such as liquified starch, soybean and cotton seed meals, corn steep, and wheat bran can lead to decrease production cost compared to chemically defined media (Tian et al., 2021). Additionally, selected candidates should preserve their viability and metabolic activities during harvesting as well as downstream processes such as air drying, freeze drying, packaging, and rehydration (Soubeyrand et al., 2006; Fleet, 2008).

Further improvement might be considered by applying the current advancements in the technology of molecular techniques, screening, and gene editing to reduce cost and improve the strains’ capabilities, however, using genetically modified organisms (GMOs) in food may raise public concerns. Developing a comprehensive understanding of the complex metabolic networks in industrial strains will enable us to develop a repertoire of genetic markers which can be used in the selection of desirable microorganisms and the development of molecular tools for improvements in the strains (Mills et al., 2010). A similar approach was used to improve LAB strains in the meat and dairy fermentation process and showed no extra risk compared to the wild strains (Mogensen, 1993; Yu et al., 2008; Xiao et al., 2010).

Finally, the Nagoya protocol should be considered before starting the development of starter cultures and their commercialization. Based on this protocol, mutually agreed terms and prior informed consent must be formed by the research provider to illustrate access to the resources and benefit shares (Johansen, 2017).

## 5. Conclusion and future perspectives

Sensory characteristics of plant-based meat analogs, including taste, aroma, color, and texture are essential components in increasing their consumer acceptability. The current approaches of making plant-based meat alternatives resemble real meat, through applying different ingredients and selection of different processes, is costly and the resulting products are far from real meat. Fermentation using different microorganisms might overcome these drawbacks and improve the final product’s sensory quality directly by creating specific ingredients more efficiently. To support the market growth of plant-based meat alternatives, further research is required to test this approach and optimize application to control its adverse impacts on plant-based meat alternatives. Applying artificial intelligence and mathematical modeling techniques might be applied to design suitable starter cultures and optimize their fermentation process more efficiently. Apart from the sensorial aspects, the impact of fermentation on the nutritional characteristic of the final products can give the plant-based meat analogs business another boost and increase its acceptability.

## Author contributions

HE: Conceptualization, Writing – original draft. XS: Writing – review & editing. RO: Supervision, Writing – review & editing. XC: Writing – review & editing. YC: Supervision, Writing – review & editing.
